# Biodistribution and radiation dosimetry of the novel hypoxia PET probe [^18^F]DiFA and comparison with [^18^F]FMISO

**DOI:** 10.1186/s13550-019-0525-6

**Published:** 2019-07-05

**Authors:** Shiro Watanabe, Tohru Shiga, Kenji Hirata, Keiichi Magota, Shozo Okamoto, Takuya Toyonaga, Kei Higashikawa, Hironobu Yasui, Jun Kobayashi, Ken-ichi Nishijima, Ken Iseki, Hiroki Matsumoto, Yuji Kuge, Nagara Tamaki

**Affiliations:** 1 Department of Nuclear Medicine, Graduate School of Medicine, Hokkaido University, Kita-15, Nishi-7, Kita-ku, Sapporo, 060-8638 Japan; 2 Division of Medical Imaging and Technology, Hokkaido University Hospital, Kita-14, Nishi-5, Kita-ku, Sapporo, 060-8648 Japan; 3 Department of Radiology, Obihiro Kosei Hospital, West 14 South 10-1, Obihiro, 080-0024 Japan; 4 Central Institute of Isotope Science, Hokkaido University, Kita-15, Nishi-7, Kita-ku, Sapporo, 060-8638 Japan; 5 Department of Pharmacy, Hokkaido University Hospital, Kita-14, Nishi-5, Kita-ku, Sapporo, 060-8648 Japan; 6 Advanced Clinical Research Center, Fukushima Global Medical Science Center, Fukushima Medical University, 1 Hikariga-oka, Fukushima, 960-1295 Japan; 7 Faculty of Pharmaceutical Sciences, Kita-14, Nishi-5, Kita-ku, Sapporo, 060-8648 Japan; 8 Research Centre, Nihon Medi-Physics Co., Ltd., 3-1 Kitasode, Sodegaura, 299-0266 Japan; 9 Department of Radiology, Kyoto Prefectural University of Medicine, Kajii-cho, Kawaramachi-Hirokoji, Kamigyo-ku, Kyoto, 602-8566 Japan

**Keywords:** Hypoxia, PET, New tracer, Dosimetry

## Abstract

**Background:**

To facilitate hypoxia imaging in a clinical setting, we developed 1-(2,2-dihydroxymethyl-3-[^18^F]-fluoropropyl)-2-nitroimidazole ([^18^F]DiFA) as a new tracer that targets tumor hypoxia with its lower lipophilicity and efficient radiosynthesis. Here, we evaluated the radiation dosage, biodistribution, human safety, tolerability, and early elimination after the injection of [^18^F]DiFA in healthy subjects, and we performed a preliminary clinical study of patients with malignant tumors in a comparison with [^18^F]fluoromisonidazole ([^18^F]FMISO).

**Results:**

The single administration of [^18^F]DiFA in 8 healthy male adults caused neither adverse events nor abnormal clinical findings. Dynamic and sequential whole-body scans showed that [^18^F]DiFA was rapidly cleared from all of the organs via the hepatobiliary and urinary systems. The whole-body mean effective dose of [^18^F]DiFA estimated by using the medical internal radiation dose (MIRD) schema with organ level internal dose assessment/exponential modeling (OLINDA/EXM) computer software 1.1 was 14.4 ± 0.7 μSv/MBq. Among the organs, the urinary bladder received the largest absorbed dose (94.7 ± 13.6 μSv/MBq). The mean absorbed doses of the other organs were equal to or less than those from other hypoxia tracers. The excretion of radioactivity via the urinary system was very rapid, reaching 86.4 ± 7.1% of the administered dose. For the preliminary clinical study, seven patients were subjected to [^18^F]FMISO and [^18^F]DiFA positron emission tomography (PET) at 48-h intervals to compare the two tracers’ diagnostic ability for tumor hypoxia. The results of the tumor hypoxia evaluation by [^18^F]DiFA PET at 1 h and 2 h were not significantly different from those obtained with [^18^F]FMISO PET at 4 h ([^18^F]DiFA at 1 h, *p* = 0.32; [^18^F]DiFA at 2 h, *p* = 0.08). Moreover, [^18^F]DiFA PET at both 1 h (*k* = 0.68) and 2 h (*k* = 1.00) showed better inter-observer reproducibility than [^18^F]FMISO PET at 4 h (*k* = 0.59).

**Conclusion:**

[^18^F]DiFA is well tolerated, and its radiation dose is comparable to those of other hypoxia tracers. [^18^F]DiFA is very rapidly cleared via the urinary system. [^18^F]DiFA PET generated comparable images to [^18^F]FMISO PET in hypoxia imaging with shorter waiting time, demonstrating the promising potential of [^18^F]DiFA PET for hypoxia imaging and for a multicenter trial.

**Electronic supplementary material:**

The online version of this article (10.1186/s13550-019-0525-6) contains supplementary material, which is available to authorized users.

## Background

There is accumulating evidence that tumor hypoxia induces the expression of gene products that confer tumor propagation, malignant progression, and broad resistance to therapy [1]. Positron emission tomography (PET) is a useful clinical tool to visualize hypoxia in vivo. Among hypoxia PET tracers, [^18^F]fluoromisonidazole ([^18^F]FMISO) is the most extensively studied; however, its optimal acquisition time is 3–4 h after injection due to its slow specific accumulation in hypoxic tissue as well as its slow clearance from the plasma [2, 3]. Consequently, next-generation tracers such as [^18^F]fluoroazomycinarabinofuranoside ([^18^F]FAZA), [^18^F]fluoroerythronitromidazole ([^18^F]FETNIM), and [^18^F]-3-fluoro-2-(4-((2-nitro-1H-imidazol-1-yl)methyl)-1H-1,2,3-triazol-1-yl)propan-1-ol ([^18^F]HX4) have been developed and applied to clinical trials [4]. Like [^18^F]FMISO, these hypoxia probes are derived from the 2-nitroimidazole in their structures. However, they still have the clinical drawbacks of poor imaging contrast at acquisition time and limited reproducibility [5, 6].

We recently developed a new imaging tracer targeting tumor hypoxia, 1-(2,2-dihydroxymethyl-3-[^18^F]-fluoropropyl)-2-nitroimidazole ([^18^F]DiFA), to overcome the disadvantages of [^18^F]FMISO and obtain better contrast image quality in a shorter period of time. [^18^F]DiFA has lower lipophilicity and is thus expected to be excreted more rapidly via the urinary system. Moreover, as another advantage, we aimed to avoid enantiomers in the structure of [^18^F]DiFA to ensure efficient synthesis and quality control, which should make hypoxia imaging more readily available for clinical application. Shimizu et al. elucidated that the mechanism by which [^18^F]DiFA and [^18^F]FMISO targets hypoxia is the same [7].

In the present study, we first evaluated the radiation dosage, biodistribution, human safety, tolerability, and early elimination of ^18^F activity in urine after the injection of a single dose of [^18^F]DiFA in healthy volunteers. We then, for the first time, compared the hypoxia detectability of [^18^F]DiFA and [^18^F]FMISO in patients with malignant tumors.

## Subjects and methods

### Healthy volunteers and patients

Eight healthy male adults (ages 21–39 years, weight 55.1–74.1 kg, Table 1) who passed the screening tests were recruited for the first-in-human study, and seven patients (ages 48–68 years, four men and three women, Table 2) were recruited for the preliminary study of [^18^F]DiFA. All seven patients had histopathologically confirmed malignant tumors which were identified in the prior imaging test.

**Table 1:** Male volunteer-specific data

No.	Age	Weight (kg)	Dose (MBq)	Mass (μg)
1	25	66.4	182.6	0.088
2	37	55.1	353.3	0.209
3	22	74.1	718.7	0.361
4	27	60.0	727.0	0.481
5	22	57.4	729.7	0.336
6	27	55.1	692.6	0.357
7	24	69.7	724.2	0.312
8	22	55.9	722.0	0.337
Mean	25.8 ± 4.7	61.7 ± 6.0	606.3 ± 200.2	0.310 ± 0.109

**Table 2:** Characteristics of the seven patients with malignant tumors

No.	Age	Sex	Weight (kg)	FMISO activity (MBq)	DiFA activity (MBq)	DiFA mass (μg)	Diagnosis
1	68	M	65	394.2	703.1	0.338	Undifferentiated pleomorphic sarcoma
2	64	F	61	384.7	714.5	0.236	Small cell lung cancer
3	56	M	60	385.6	715.6	0.237	Rectal adenocarcinoma
4	63	F	51	374.6	725.2	0.277	Tongue squamous cell carcinoma
5	64	M	73	386.5	716.9	0.274	Liposarcoma
6	65	M	76	387.4	716.4	0.299	Hepatocellular carcinoma
7	48	F	52	386.5	733.3	0.306	Malignant melanoma
Mean	61.1 ± 6.3		62.6 ± 8.9	385.6 ± 5.4	717.9 ± 8.7	0.281 ± 0.034	

### Radiopharmaceutical preparation

**Fig. 1:**
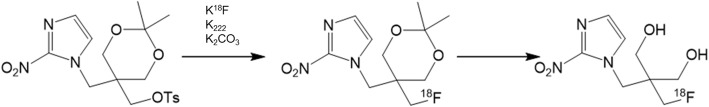
Synthesis of [^18^F]DiFA from its precursor

[^18^F]DiFA was prepared by the nucleophilic fluorination of 2,2-dimethyl-5-[2-(2-nitro-1H-imidazole-1-yl)ethyl]-5-(p-toluenesulfonyloxymethyl)-1,3-dioxane followed by acidic hydrolysis of the protecting group using an automated synthesis apparatus (UG-M1; Universal Giken, Odawara, Japan) (Fig. 1, Additional file 1: Table S1).

[^18^F]FMISO was prepared by the nucleophilic fluorination of the precursor molecule 1-(2′-nitro-1′-imidazolyl)-2-O-tetrahydropyranyl-3-O-toluenesulphonylpropanediol in a manner similar to that for [^18^F]DiFA using previously reported procedures [8, 9].

The product specifications of radiochemical purity were set to be > 95% for [^18^F]DiFA and [^18^F]FMISO in accordance with the previous report on [^18^F]FMISO [10]. The actual radiochemical purity used in the present study was 99.0 ± 1.0% for [^18^F]DiFA and > 95% for [^18^F]FMISO.

### Biodistribution, dosimetry, and safety of [^18^F]DiFA in the healthy volunteers

**Fig. 2:**
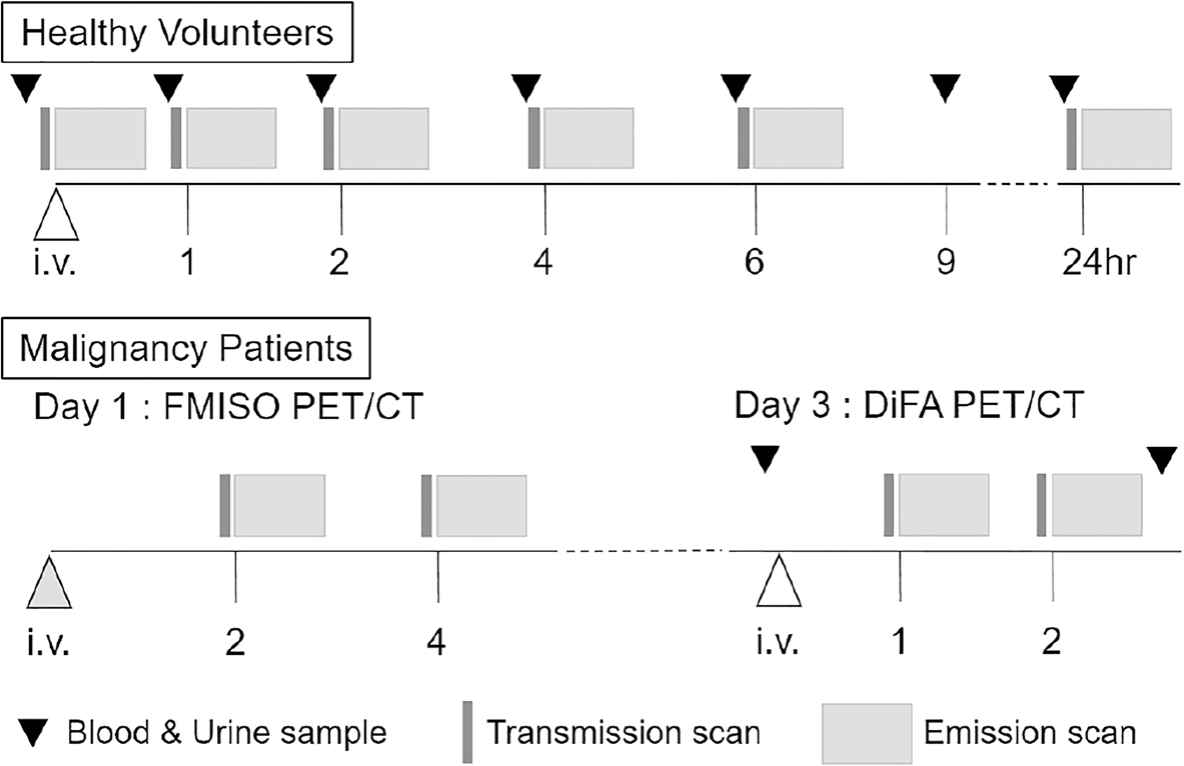
The protocol of the dosimetry study of [^18^F]DiFA (upper) and comparative study with [^18^F]DiFA and [^18^F]FMISO PET/CT imaging

The biodistribution and dosimetry procedure are shown in Fig. 2. [^18^F]DiFA was intravenously injected over a 1-min period. We increased the injected activity in a step-by-step manner. Volunteers #1 and #2 were injected with 185 MBq and 370 MBq of [^18^F]DiFA, respectively, following approval by a third-party safety committee. The other six volunteers (#3–#8) were injected with 740 MBq of [^18^F]DiFA.

After the injection, the emission study was started with a 5-min dynamic scanning with the field of view (FOV) containing the entire heart, followed by serial three-bed scanning covering from the heart to the kidneys until 60 min post-injection, on a Gemini TF PET/computed tomography (CT) scanner (Philips Healthcare, Cleveland, OH). After the dynamic scanning, five serial whole-body PET scans were acquired at 1, 2, 4, 6, and 24 h post-injection. Prior to each emission imaging session, a whole-body low-dose CT image was acquired for attenuation and scatter corrections. The whole-body PET scans were acquired in 3D mode and ranged from the vertex to the toes. The duration of emission scanning was 1.5 min per bed position.

The PET images were reconstructed using 3-dimensional blob-based iterative list-mode ordered-subsets expectation maximization (OSEM) algorithm with time-of-flight information, following default settings: iterations, 3; subsets, 33; blob increment, 2.0375 voxels; blob radius, 2.5 voxels; blob shape parameter alpha, 8.3689; and relaxation parameter, 0.7. The image matrix size was 144 × 144 pixels for the 576-mm FOV, and the voxel size was 4 × 4 × 4 mm^3^. The reconstruction included corrections for normalization, dead time, attenuation, scatter, random coincidences, sensitivity, and decay. The reconstructed images were not additional post-filtered.

PET images for volunteers #3–#8 were analyzed using PMOD software ver. 3.1 (PMOD Technologies, Zurich, Switzerland). The uptake of each major organ was calculated by drawing a volume of the region based on the contours of the PET and CT images as described previously [11]. At each time point, the decayed radioactivity of each source organ is expressed as a percentage of the injected activity (%IA) and plotted against time, and fitted to an exponential or sum-of-exponentials function in organ level internal dose assessment/exponential modeling (OLINDA/EXM) 1.1 computer software (Vanderbilt University, Nashville, TN) [12] to determine the total number of disintegrations per unit of administered activity, hereafter referred to as the normalized number of disintegrations.

Urine samples were collected for the following intervals: 0–1 h, 1–2 h, 2–4 h, 4–6 h, 6–9 h, and 9–24 h. The urine samples were assayed for radioactivity with an auto-well gamma counter (ARC-400; Hitachi, Tokyo) to estimate the total excreted activity. The residence time of the urinary bladder was also determined using the dynamic bladder model in OLINDA/EXM 1.1 as described previously [11].

Voiding intervals were set to 2 h to calculate the dose estimates for the urinary bladder wall. The effective doses in the various organs were calculated by entering the normalized number of disintegrations of all source organs for each subject into OLINDA/EXM, using the standardized adult male models. Data are shown as the mean ± standard deviation.

The subjects’ blood pressure, body temperature, pulse rate, oxygen saturation, blood, and urine sampling were monitored before the administration of [^18^F]DiFA and following all PET/CT scans in addition to 9 h after the administration of [^18^F]DiFA.

### Patient study

All seven patients underwent both [^18^F]DiFA and [^18^F]FMISO scans with a 2-day interval. All patients were injected with up to 740 MBq of [^18^F]DiFA and 400 MBq of [^18^F]FMISO. PET scans were acquired at 1 and 2 h post-injection, and [^18^F]FMISO PET scans were acquired at 2 and 4 h post-injection. The reconstruction method was the same as that used with the whole-body PET scanning in this first-in-humans study, and the scanning of the lesion was performed for 10 min.

All PET/CT images were independently visually interpreted using XTREK software (J-MAC Systems, Sapporo, Japan) by two experienced certified specialists in nuclear medicine (SW and KH). The observers were blinded both to the tracer and to the timing of the scan. The observers used both gray and color scales where the upper and lower limits could be changed manually. The tracer uptake in the lesions over 10 mm in size was assessed and assigned an intensity uptake score of 0–4 as follows: 0, uptake less than background; 1, no regions of focal uptake greater than background; 2, focal uptake mildly greater than background; 3, focal uptake moderately greater than background; and 4, focal uptake markedly greater than background. The normal area of the same organ where the tumor existed, or normally-appearing muscle if the tumor existed in fatty tissues such as retroperitoneum, was chosen as the background. As the next step, the lesions were grouped into two classes: those with uptake scores of ≥ 2 were considered hypoxia-positive and the others were deemed hypoxia-negative.

Histopathological confirmation was not practical in all of the lesions. The lesions were considered positive when the 4-h [^18^F]FMISO PET image was positive, based on the general consensus that [^18^F]FMISO is the gold standard in clinical research for the measurement of hypoxia. Cases of disagreement were resolved by consensus.

The inter-observer agreement regarding the specialists’ visual analysis and the hypoxia positivity of the lesions was evaluated using kappa values. The sensitivity, specificity, and accuracy of hypoxia were evaluated in the [^18^F]DiFA PET.

The quality of the agreements was defined as follows, according to the Cohen *k* test: 0–0.2, poor agreement; 0.21–0.40, fair agreement; 0.41–0.60, moderate agreement; 0.61–0.80, good agreement; and 0.81–1.00, very good agreement. For each imaging protocol, diagnostic performances (sensitivity, specificity, and accuracy) were assessed and compared using the McNemar test. Statistical calculations were carried out using JMP® 14 software (SAS, Cary, NC, USA).

## Results

### Safety of [^18^F]DiFA

None of the eight volunteers presented with any symptoms or clinically detectable adverse pharmacological effects. No significant changes in vital signs or the results of laboratory tests were observed during the first 24-h observation period following the tracer administration or the follow-up visit at 1 week (Additional file 2). The radiation exposure of attenuation correction CT in volunteers was totally 43.0 ± 0.9 mSv.

### Imaging and biodistribution of [^18^F]DiFA

**Fig. 3:**
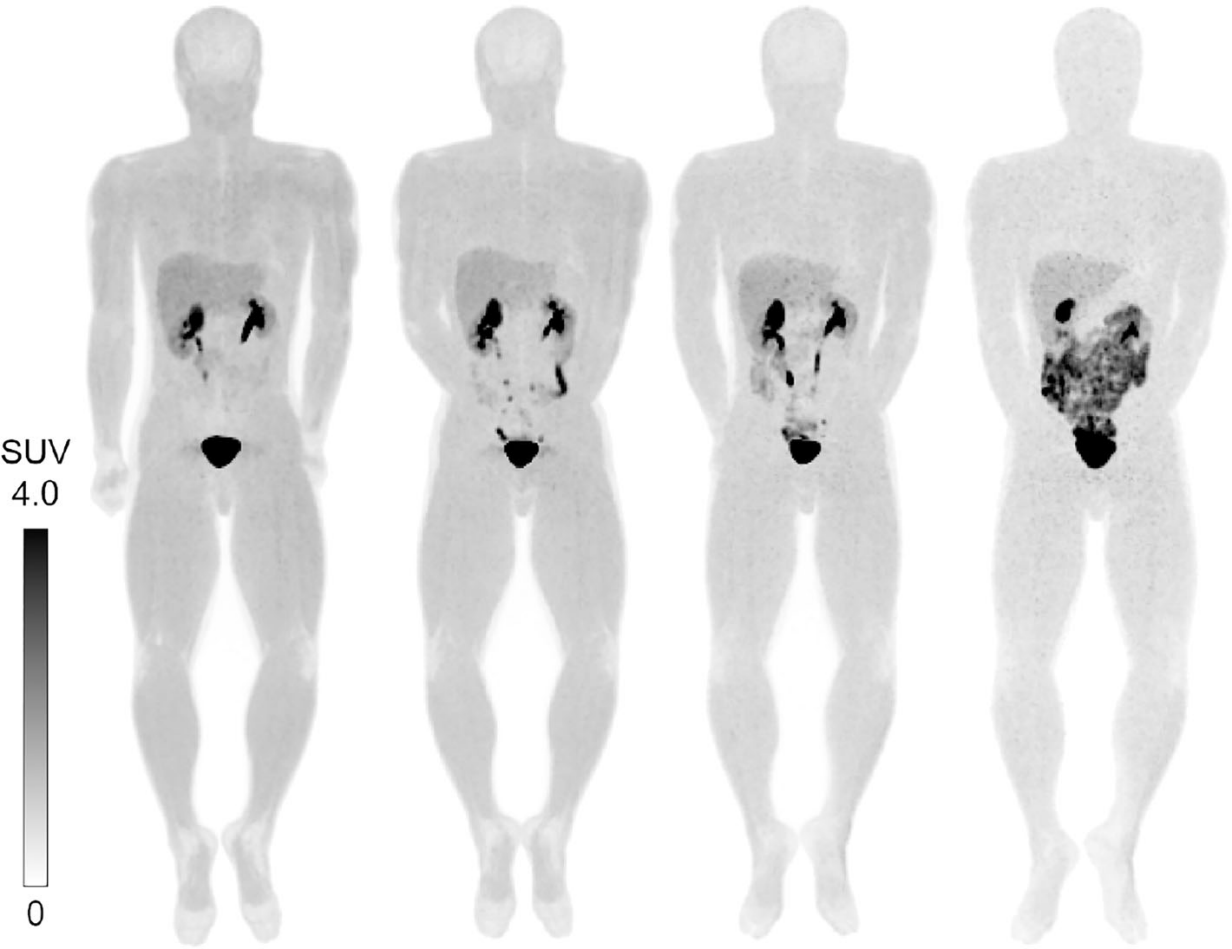
MIP images of [^18^F]DiFA in a healthy volunteer. Decay-corrected anterior maximum-intensity projections of PET at 1, 2, 4, and 6 h (from left to right) after an injection of 718.7 MBq of [^18^F]DiFA in a 74.0-kg male volunteer. There was a rapid clearance of activity in the kidneys, liver, and bladder. Gallbladder activity peaked following the first meal after the PET acquisition at 4 h, then decreased with time

The main characteristics of the radiotracer uptake are illustrated in PET maximum intensity projection (MIP) images for one of the normal volunteers from the PET scans in Fig. 3. In the early scans, a predominant accumulation was observed in the subjects’ urinary tract (the renal pelvis, ureter, and bladder) with moderate uptake in the liver. The gallbladder and large intestine showed strong radioactivity in later scans. The brain showed no significant uptake (even lower than muscles), reflecting the hydrophilic characteristics of [^18^F]DiFA. All other organs showed background levels of activity.

**Fig. 4:**
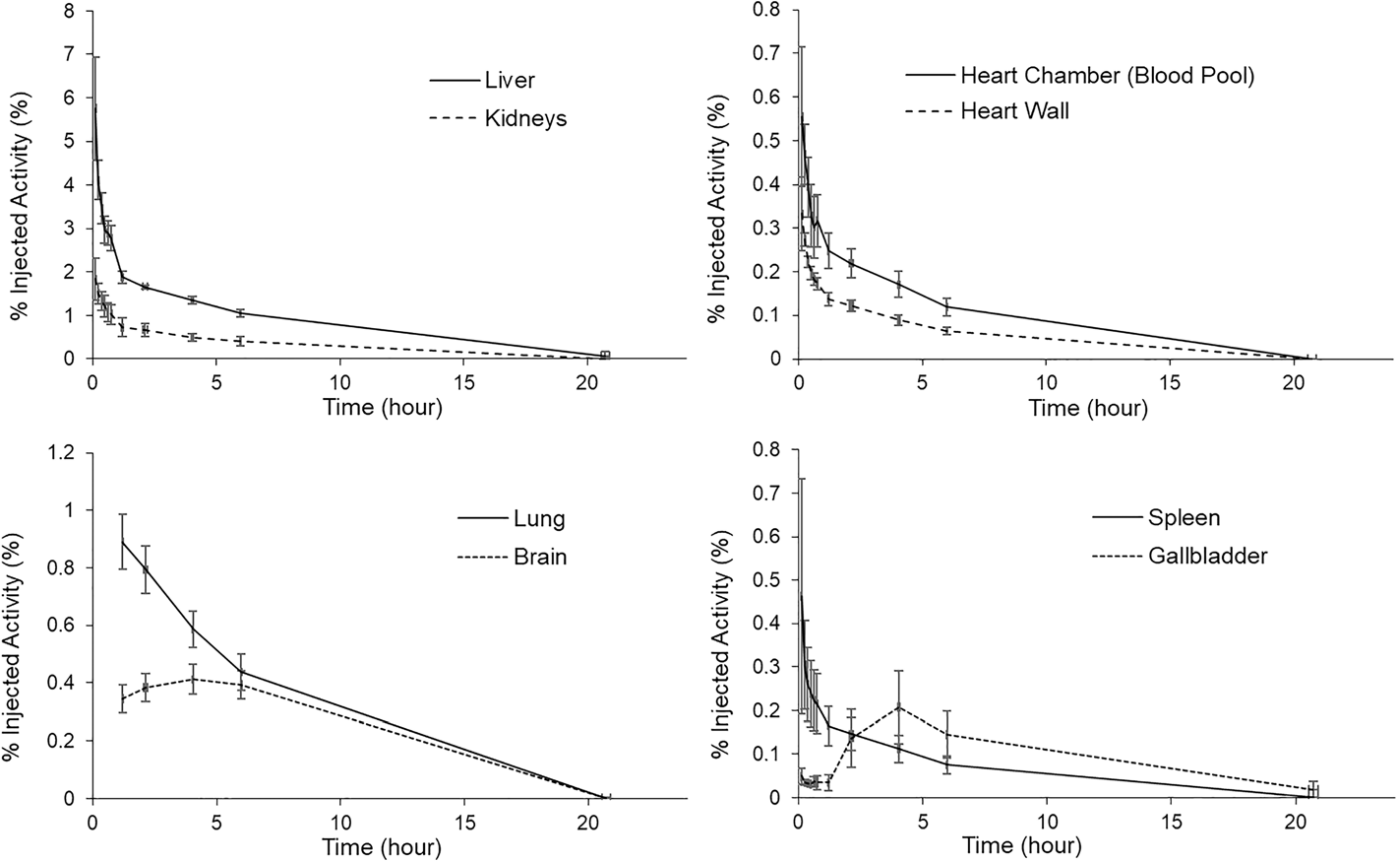
Time-activity curves in representative organs. [^18^F]DiFA was rapidly cleared from the organs. The accumulation in the gallbladder peaked after 4 h, since [^18^F]DiFA that was excreted in the bile due to hepatobiliary excretion stayed in the gallbladder and was secreted with a meal (at 4 h after examination). The accumulation in the brain slowly increased, reflecting the improvement in water solubility, and after 4 h, it peaked and then decreased. The data are the mean ± SD of results from six volunteers

**Fig. 5:**
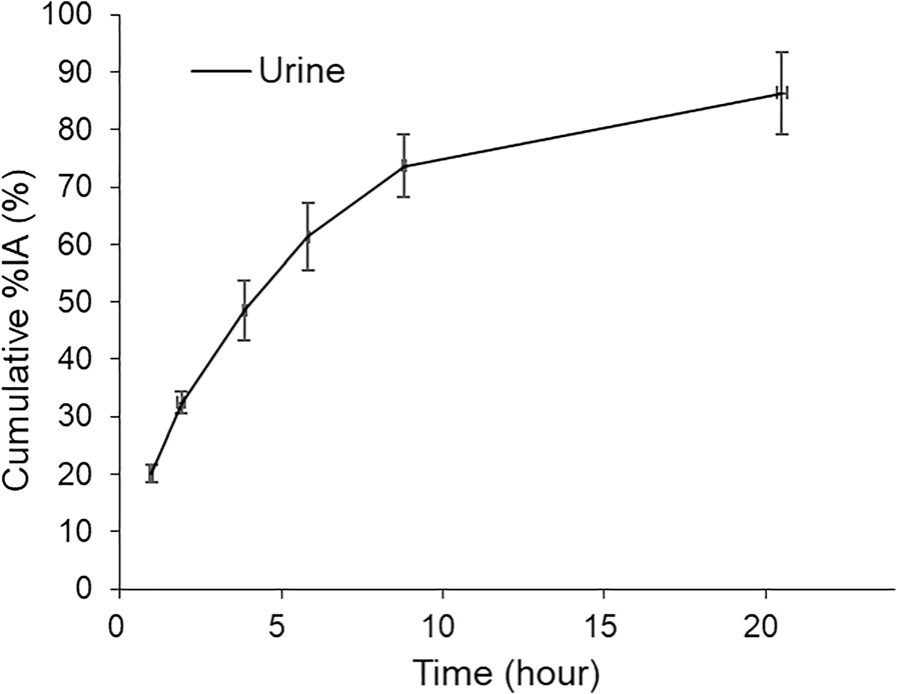
[^18^F]DiFA in urine expressed as a % of injected activity (%IA)

In all eight subjects, the highest (decay-corrected) uptake of [^18^F]DiFA was found in the muscle, with peak values of 26.2 ± 3.4% injected activity (IA), followed by the liver at 5.7 ± 1.2% IA. The %IA decreased gradually toward the end of the study in all organs except for the brain and gallbladder (Fig. 4). Although the radioactivity of [^18^F]DiFA was excreted primarily via the renal system, a small portion of [^18^F]DiFA was excreted via the bile system. By the end of the study (20–24 h), approx. 86.4 ± 7.1% of the injected activity of [^18^F]DiFA had been excreted in the urine (Fig. 5). This clearance was relatively rapid among hypoxia tracers, with 20.2 ± 1.6% of the tracer being excreted by 1 h after the injection.

### Dosimetry of [^18^F]DiFA

The mean residence times for the organs are listed in Table 3. The mean effective dose of [^18^F]DiFA was estimated as 14.4 ± 0.7 μSv/MBq for the 2-h bladder-voiding models. The highest radiation-equivalent dose was calculated for the urinary bladder wall, with 0.09 mSv/MBq (Table 4).

**Table 3:** Residence times of [^18^F]DiFA for measured source organs

Brain	0.009 ± 0.001
Gallbladder contents	0.003 ± 0.001
Lower large intestine	0.002 ± 0.000
Small intestine	0.024 ± 0.004
Upper large intestine	0.013 ± 0.002
Heart contents	0.007 ± 0.001
Heart wall	0.004 ± 0.000
Kidneys	0.021 ± 0.004
Liver	0.057 ± 0.004
Lungs	0.020 ± 0.002
Muscle	0.542 ± 0.052
Pancreas	0.003 ± 0.001
Red marrow	0.098 ± 0.013
Spleen	0.005 ± 0.002
Remainder	1.066 ± 0.104
Urinary bladder contents (2 h)	0.184 ± 0.027

The data are mean ± SD (hour), *n* = 6 patients

**Table 4:** Equivalent doses of [^18^F]DiFA, [^18^F]FMISO, [^18^F]FAZA, [^18^F]HX4, and [^18^F]FETNIM to target organs

Target organs	Tracer				
	[^18^F]DiFA (2-h void)	[^18^F]FMISO [13] (2-h void)	[^18^F]FAZA [11] (2-h void)	[^18^F]HX4 [14] (1-h void)	[^18^F]FETNIM [15] (2-h void)
Adrenals	0.009 ± 0.000	0.017	0.012 ± 0.001	0.009 ± 0.001	0.012
Brain	0.004 ± 0.000	0.009	0.004 ± 0.001	0.005 ± 0.001	0.006
Breasts	0.006 ± 0.000	0.012	0.009 ± 0.001	0.006 ± 0.001	0.007
Gallbladder wall	0.014 ± 0.002	0.015	0.013 ± 0.004	0.024 ± 0.007	0.014
LLI wall	0.013 ± 0.001	0.014	0.013 ± 0.006	0.022 ± 0.002	0.012
Small intestine	0.015 ± 0.001	0.013	0.012 ± 0.005	0.015 ± 0.000	0.012
Stomach wall	0.009 ± 0.000	0.013	0.012 ± 0.001	0.009 ± 0.001	0.012
ULI wall	0.015 ± 0.001	0.014	0.013 ± 0.002	0.016 ± 0.003	0.014
Heart wall	0.008 ± 0.000	0.019	0.018 ± 0.001	0.009 ± 0.001	0.011
Kidneys	0.018 ± 0.003	0.016	0.017 ± 0.002	0.019 ± 0.002	0.027
Liver	0.011 ± 0.001	0.018	0.016 ± 0.003	0.014 ± 0.003	0.024
Lungs	0.008 ± 0.000	0.010	0.011 ± 0.008	0.008 ± 0.001	0.008
Muscle	0.008 ± 0.000	0.014	0.011 ± 0.009	0.008 ± 0.001	0.012
Ovaries	0.012 ± 0.000	0.018	0.014 ± 0.001	0.012 ± 0.001	0.013
Pancreas	0.012 ± 0.002	0.018	0.013 ± 0.002	0.010 ± 0.001	0.019
Red marrow	0.014 ± 0.001	0.011	0.011 ± 0.001	0.008 ± 0.001	0.012
Osteogenic cells	0.014 ± 0.000	0.008	0.011 ± 0.002	0.012 ± 0.001	0.011
Skin	0.006 ± 0.000	0.005	0.008 ± 0.001	0.006 ± 0.001	0.007
Spleen	0.010 ± 0.002	0.016	0.017 ± 0.008	0.009 ± 0.001	0.020
Testes	0.009 ± 0.000	0.015	0.004 ± 0.001	0.009 ± 0.001	0.010
Thymus	0.007 ± 0.000	0.016	0.011 ± 0.003	0.008 ± 0.001	0.009
Thyroid	0.007 ± 0.000	0.015	0.010 ± 0.001	0.008 ± 0.001	0.009
Urinary bladder wall	0.095 ± 0.014	0.021	0.047 ± 0.008	0.085 ± 0.010	0.062
Uterus	0.015 ± 0.001	0.018	0.020 ± 0.001	0.014 ± 0.001	0.015
Total body	0.009 ± 0.000	0.013	0.012 ± 0.002	0.008 ± 0.001	0.011
Effective dose	0.014 ± 0.001	0.013	0.013 ± 0.004	0.014 ± 0.001	0.015

*LLI* lower large intestine, *ULI* upper large intestine. The data are mean ± SD/mean, mSv/MBq, *n* = 6 subjects.

### Comparison of [^18^F]DiFA and [^18^F]FMISO in patients

The seven patients had 19 lesions that could be evaluated. The radiation exposure of attenuation correction CT and FMISO was 2.9 ± 1.5 mSv and 5.0 ± 0.1 mSv, respectively.

The visual analyses of [^18^F]DiFA PET at both 1 h and 2 h compared to [^18^F]FMISO at 4 h are shown in Table 5. Table 6 shows the global agreements of the visual analyses of [^18^F]FMISO and [^18^F]DiFA PET/CT images. The inter-observer agreement of the five-level grading was better for [^18^F]DiFA (*κ* = 0.60 at 1 h, 1.00 at 2 h) than for [^18^F]FMISO (*κ* = 0.29 at 2 h, 0.38 at 4 h) in this study.

**Table 5:** Performance of [^18^F]DiFA in detecting hypoxia compared to [^18^F]FMISO at 4 h

	Observer 1		Observer 2		Observer 1 and 2	
	[^18^F]DiFA (1 h (+))	[^18^F]DiFA (1 h (−))	[^18^F]DiFA (1 h (+))	[^18^F]DiFA (1 h (−))	[^18^F]DiFA (2 h (+))	[^18^F]DiFA (2 h (−))
[^18^F]FMISO at 4 h (+)	9	0	8	1	9	0
[^18^F]FMISO at 4 h (−)	1	9	3	7	3	7

**Table 6:** *κ* value for PET/CT images

*κ*	[^18^F]FMISO		[^18^F]DiFA	
	2 h	4 h	1 h	2 h
Five classes	0.29	0.63	0.60	1.00
Two classes	0.38	0.59	0.68	1.00

**Fig. 6:**
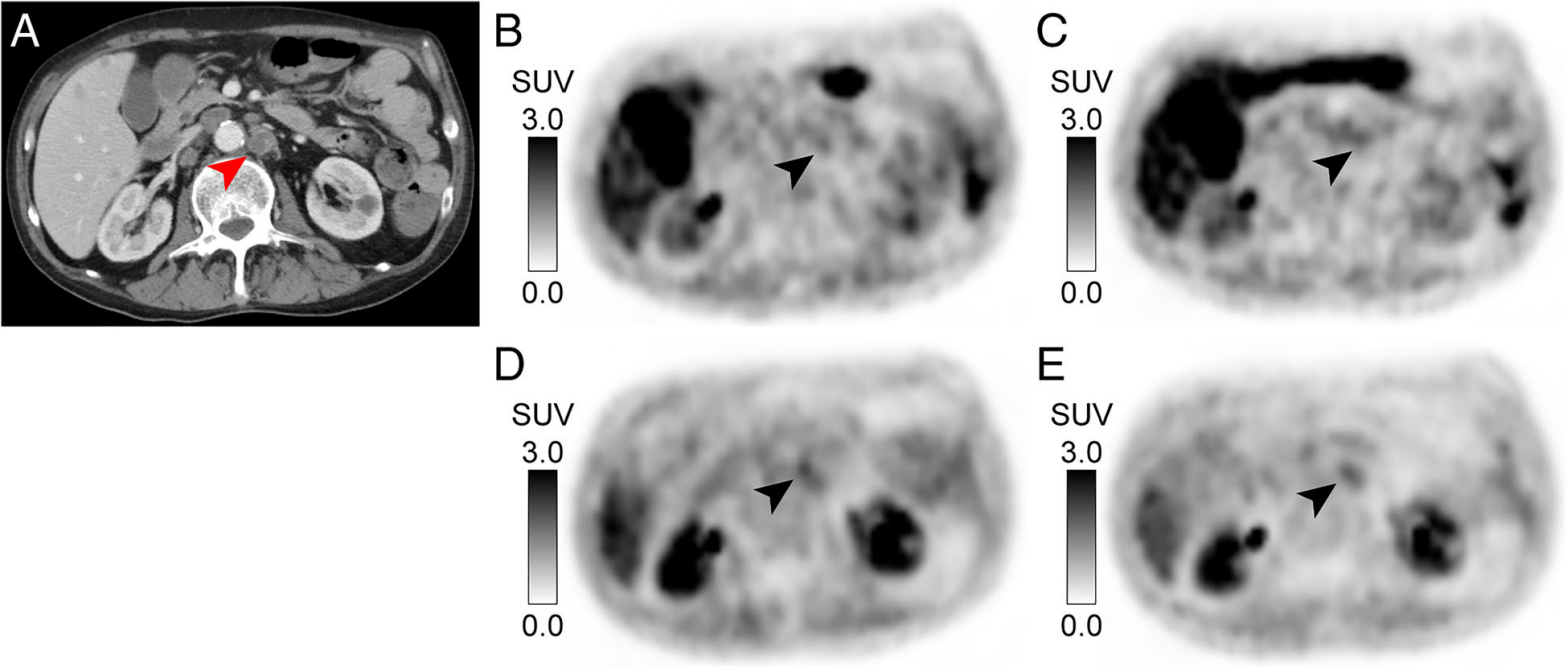
Representative images of a patient. **a** Transaxial image of contrast-enhanced CT. The swollen paraaortic lymph node is shown. **b**, **c** 2-h and 4-h images with [^18^F]FMISO. **d**, **e** 1-h and 2-h images with [^18^F]DiFA. Tracer uptake to the lymph node metastasis of rectal adenocarcinoma is clearly detected in all images. The contrast at 1 h and 2 h with [^18^F]DiFA (TMR = 1.25 and 1.37, respectively) is better than that at 2 h with [^18^F]FMISO (TMR = 1.09) and equivalent to 4-h [^18^F]FMISO (TMR = 1.44)

The diagnostic performance [^18^F]DiFA is summarized in Table 7. There was no significant difference in the detection of tumor hypoxia between [^18^F]FMISO and [^18^F]DiFA (Fig. 6).

**Table 7:** Diagnostic accuracy of [^18^F]DiFA PET/CT for the detection of tumor hypoxia

[^18^F]DiFA 1 h					[^18^F]DiFA 2 h			
Observer	Sensitivity	Specificity	Accuracy	*p* value*	Sensitivity	Specificity	Accuracy	*p* value*
No. 1	1.00	0.90	0.95	0.32	1.00	0.70	0.84	0.08
No. 2	0.89	0.70	0.79	0.32	1.00	0.70	0.84	0.08

*McNemar’s test

## Discussion

In this work, we first evaluated the safety and dosimetric data of [^18^F]DiFA, a new [^18^F]FMISO-based derivative with stronger hydrophilicity, in healthy volunteers for its potential use as a hypoxia PET tracer. We found that [^18^F]DiFA caused no adverse effects after injection and had rapid clearance from the urine and reasonable biodistribution and dosimetry profiles in human subjects. In addition, in our comparison of the abilities of [^18^F]DiFA and [^18^F]FMISO to diagnose tumor hypoxia, we found that the diagnostic abilities were approximately equivalent, and good inter-observer reproducibility was observed even though there was a shorter time from the injection to the scan with the use of [^18^F]DiFA.

In our human study, high [^18^F]DiFA uptake/excretion was observed in the liver, muscle, and kidneys, whereas low uptake was observed in the lung and brain. These findings are similar to those for other hypoxia tracers, and the usefulness of evaluating neoplasms in the cranial should be investigated in detail. [^18^F]DiFA clearance from the blood pool is very rapid, and the accumulation in the blood pool was obscured at just 1 h after administration. In addition, since the distributions in the liver and intestines were relatively low, patients with hepatobiliary and colorectal cancers may be good candidates for hypoxia imaging using [^18^F]DiFA if the images are acquired at an appropriate time point.

[^18^F]DiFA had a higher rate of urinary excretion (86.4%) than other hypoxia tracers, such as [^18^F]FAZA (15% [11]), [^18^F]FETNIM (60% [15]), and [^18^F]HX4 (45% at 3.6 h [14]). In general, more hydrophilic compounds will have more rapid biodistribution and clearance from the body [16]. [^18^F]DiFA may thus be the ideal tracer for hypoxia imaging. In a comparison of our present dosimetry data with the available literature on [^18^F]FMISO [13], [^18^F]FAZA [11], [^18^F]HX4 [14], and [^18^F]FETNIM [15], radiation dose of these tracers resulted in almost the same distribution as that of [^18^F]DiFA. Although, in the case of [^18^F]DiFA, the radiation dose to the bladder wall is increased due to the high water solubility of this tracer, it is expected that the actual radiation dose can be reduced by shortening the urinary excretion interval (i.e., frequent voiding). No acute radiation toxicity was expected with 740 MBq F-18. Also, no chemical toxicity was expected with the unlabeled part of the 0.310 ± 0.109 μg DiFA since the maximum non-toxic dose was 517 μg/kg based on our preclinical study. We confirmed that there was no toxicity based on the laboratory tests.

To assess the potential of [^18^F]DiFA as a specific PET tracer for hypoxia imaging, we examined seven patients with malignant tumors by both [^18^F]FMISO and [^18^F]DiFA PET/CT imaging. In this study, there was a 48-h interval between [^18^F]FMISO and [^18^F]DiFA imaging. Hypoxia is an unstable condition that can be altered in a short time, theoretically explained by acute and chronic hypoxia. In our previous clinical study using [^18^F]FMISO, however, we confirmed that the degree and localization of hypoxia were stable between 2 scans of [^18^F]FMISO-PET with 48 h interval [17]. Therefore, we assumed that the hypoxia status was stable between [^18^F]FMISO and [^18^F]DiFA imaging. The results showed that there were no significant differences between [^18^F]FMISO and [^18^F]DiFA PET for the detection of tumor hypoxia. Moreover, [^18^F]DiFA PET at both 1 and 2 h post-injection showed better inter-observer reproducibility than [^18^F]FMISO at 4 h. Because [^18^F]FMISO has characteristics of slow accumulation and slow clearance from blood and normal tissues, background organs often show high accumulation when imaged, resulting in a low tumor-to-background ratio (Fig. 6). Early scans are greatly affected by perfusion and degrade accuracy and reproducibility. However, the image quality was poor in [^18^F]FMISO PET image 4 h after injection due to > 2× half-life of F-18. We think these are the main reasons for degraded inter-operator reproducibility for [^18^F]FMISO PET. In contrast, [^18^F]DiFA is rapidly cleared from the blood and normal tissues, enhancing lesion-to-background contrast. In addition, early scan timing keeps a high signal-to-noise ratio, which leads to good image quality. The rapid clearance of [^18^F]DiFA provided an easy evaluation and high reproducibility. From our results, 4 h [^18^F]FMISO PET has considered the reference standard in clinical and [^18^F]DiFA PET showed 3 false positives out of 10 lesions. The disparity between the two tracer datasets may be mainly due to the pharmacokinetics of the tracer itself, but also partly by the possible fluctuation of hypoxic status (so-called, acute hypoxia).

The tracer activity was determined by our preclinical study of dose exposure using rat. The activity of 740 MBq of [^18^F]DiFA was acceptable for the first-in-man study as maximum activity, and we confirmed its safety in healthy subjects and cancer patients. From this dataset, we will determine the optimal activity of [^18^F]DiFA. Wang et al. [18] and Thorwarth et al. [19] reported that the kinetic model of [^18^F]FMISO PET was the 3-compartment model. The pharmacokinetic model of [^18^F]DiFA is considered the same as for [^18^F]FMISO. In these tracers, although the injected activity of the [^18^F]DiFA was almost double that of [^18^F]FMISO, the lesion-to-background ratio did not change with increased activity (Additional file 3). [^18^F]DiFA has the advantage of early scan time for reducing patients waiting time, easy synthesis for robust results, and good reproducibility for the assessment of hypoxia status in multicenter trials. In the present investigation, the SUVmax values obtained with [^18^F]DiFA were lower than those obtained with [^18^F]FMISO, in agreement with a preclinical study by Yasui et al. [20].

The most important limitation of [^18^F]FMISO is its high lipophilicity, which causes slow tracer accumulation, slow plasma clearance, and low tumor-to-background contrast [2]. Our group showed that [^18^F]FMISO PET for hypoxia imaging achieved better quality at 4 h compared to 2 h, with a better lesion-to-background ratio [21], better differential diagnoses between glioblastoma and lower-grade gliomas at 4 h [22] than at 2 h [23], and high test-retest reproducibility of the tracer distribution at 4 h [17]. In this study, thus, we set the 4-h after injection of [^18^F]FMISO as the gold standard.

New-generation hypoxia tracers have been developed to improve hydrophilicity and accelerate the tracers’ clearance from normal oxygenated tissues [24]. A previous report using a single murine xenograft tumor model condition showed [^18^F]FMISO, [^18^F]FAZA, and [^18^F]HX4 demonstrated similar tumor distributions, and highest TBRs for [^18^F]FAZA and [^18^F]HX4 were obtained at 2 h p.i. and 3 h p.i., respectively. [^18^F]FMISO and [^18^F]DiFA did not show plateau formation and had better TBR at later time points [24, 25]. There is no clinical study which compared the uptake between [^18^F]FAZA and [^18^F]FMISO, while [^18^F]HX4 imaging in head and neck cancer patients at 1.5 h p.i. was found to have TMR properties similar to those of [^18^F]FMISO at 2 h p.i. Wei et al. reported that [^18^F]FMISO showed significantly higher uptake than [^18^F]FETNIM in tumor/non-tumor ratio in lung cancer [26]. More data have been accumulated for [^18^F]FAZA than for any other second-generation hypoxia tracers, but the reproducibility of the scans using [^18^F]FAZA has not been established. While a preclinical micro-PET analysis showed voxel-to-voxel reproducibility between two baseline scans performed 24 h apart, another preclinical report in an animal tumor model showed that [^18^F]FAZA uptake was less reproducible after 48 h, even without additional anticancer treatment [5]. There has been no report about the reproducibility of [^18^F]FAZA in a clinical trial. In our preclinical research using ex vivo autoradiography, the uptake of [^18^F]DiFA, formerly called HIC101, was shown to have a significant positive correlation with regions of pimonidazole distribution, indicating that [^18^F]DiFA was selectively accumulated in tumor hypoxic regions [20, 27]. Yasui et al. reported that the tumor-to-muscle ratios were significantly higher as early as at 1 h post-injection. In the current study, the images of 1 h p.i. of [^18^F]DiFA and the images of 4 h p.i. of [^18^F]FMISO were equivalent, indicating the potential to obtain an image with good contrast at 1 h after injection.

Masaki et al. [28] and Shimizu et al. [7] described the mechanisms of [^18^F]FMISO and [^18^F]DiFA. In brief, the glutathione-conjugated reductive metabolites of the tracer are present in the hypoxic regions of tumor tissues, suggesting that the tracer undergoes the glutathione conjugation reaction following reductive metabolism in hypoxic cells. The glutathione conjugate of reduced tracer was the major metabolite involved in the hypoxia-specific accumulation. In our preclinical study, while [^18^F]DiFA accumulated in hypoxic cells was higher than that of [^18^F]FMISO, [^18^F]DiFA was rapidly cleared from the blood [7]. When administered to mice, the tumor-to-blood and the tumor-to-muscle ratios of [^18^F]DiFA were higher than those of [^18^F]FMISO. The rapid clearance may have contributed to the reduced non-specific accumulation of [^18^F]DiFA in the background tissues. Therefore, the distribution of [^18^F]DiFA may be equally or highly specific to the hypoxic region of tumor tissues compared to that of [^18^F]FMISO [7]. In the current human study, the background of [^18^F]DiFA tended to be lower compared to that of [^18^F]FMISO (Additional file 4: Figure S1). Thus, [^18^F]DiFA achieved better contrast imaging of tumor hypoxia with a shorter waiting time compared to [^18^F]FMISO via the same mechanism.

The major limitations of this preliminary study were that only seven patients were enrolled for the diagnostic assessment of hypoxia detection by [^18^F]FMISO and [^18^F]DiFA and the primary tumors or metastases were varied, rather than restricted to a specific type of lesion. We plan to conduct verification studies on specific carcinomas soon. Since [^18^F]DiFA undergoes the same metabolic pathway of [^18^F]FMISO in hypoxic cells, the distribution of [^18^F]DiFA in tumor tissues might be affected by factors related to glutathione conjugation. Previously, our group investigated the mechanisms of the [^18^F]FMISO uptake in three cell lines (FaDu, LOVO, and T24) and found that [^18^F]FMISO accumulation was associated with glutathione conjugation ability as well as hypoxic conditions [29]. Similarly, [^18^F]DiFA accumulation might be affected by cellular factors such as the cellular reduced glutathione level and glutathione S-transferases, which catalyze glutathione conjugation reactions, and the multidrug resistant protein 1, which exports various kinds of glutathione conjugates out of cells. Therefore, further preclinical and clinical studies are needed to clarify whether these factors actually influence the imaging of [^18^F]DiFA. In addition, we studied whether [^18^F]FMISO and [^18^F]DiFA exhibit the same distribution 48 h apart, but we did not compare [^18^F]DiFA and new hypoxia tracers such as [^18^F]FAZA or [^18^F]HX4. Further research on the reproducibility of [^18^F]DiFA findings is also necessary.

## Conclusions

The current study revealed 2 important findings. First, [^18^F]DiFA is well tolerated and its radiation dose is comparable to that of [^18^F]FMISO and other hypoxia tracers. [^18^F]DiFA showed very rapid clearance and a large fraction of renal excretion. Second, [^18^F]DiFA achieved an equivalent image quality compared with [^18^F]FMISO, with smaller inter-observer variability. Thus, [^18^F]DiFA PET enables hypoxia imaging with equivalent contrast in shorter waiting time and would be potentially suitable for a multicenter trial.

## Additional files

Additional file 1: **Table S1.** Reaction sequence for [^18^F]DiFA radiosynthesis. (DOCX 15 kb)

Additional file 2: The results of laboratory tests. (XLSX 33 kb)

Additional file 3: The lesion-to-background ratio formula. (DOCX 30 kb)

Additional file 4: **Figure S1.** [^18^F]DiFA vs. [^18^F]FMISO (PDF 736 kb)

## Data Availability

The datasets used and/or analyzed during the current study are available from the corresponding author on reasonable request.

## Abbreviations

[^18^F]DiFA: 1-(2,2-dihydroxymethyl-3-[^18^F]-fluoropropyl)-2-nitroimidazole
[^18^F]FAZA: [^18^F]Fluoroazomycinarabinofuranoside
[^18^F]FETNIM: [^18^F]Fluoroerythronitromidazole
[^18^F]FMISO: [^18^F]Fluoromisonidazole
[^18^F]HX4: [^18^F]-3-Fluoro-2-(4-((2-nitro-1H-imidazol-1-yl)methyl)-1H-1,2,3-triazol-1-yl)propan-1-ol
CT: Computed tomography
FOV: Field of view
IA: Injected activity
MIP: Maximum intensity projection
MIRD: Medical internal radiation dose
OLINDA/EXM: Organ level internal dose assessment/exponential modeling
OSEM: Ordered-subsets expectation maximization
PET: Positron emission tomography
