# Supplementary material for: Biodistribution and radiation dosimetry of the novel hypoxia PET probe [18F]DiFA and comparison with [18F]FMISO
Source: EJNMMI Res. 2019 Jul 5;9:60. doi: 10.1186/s13550-019-0525-6 (PMC6611855; doi:10.1186/s13550-019-0525-6)
Supplement: Supplementary file 1 — Table S1. Reaction sequence for [18F]DiFA radiosynthesis. (DOCX 15 kb) [file 13550_2019_525_MOESM1_ESM.docx]

| **Additional file 1: Table S1**. Reaction sequence for [^18^F]DiFA radiosynthesis | |
| --- | --- |
| 01. ^18^F trapping on QMA cartridge |  |
| 02. ^18^F elution with K2.2.2/K_2_CO_3_ solution |  |
| 03. Azeotropic evaporation of the solvent |  |
| 04. Addition of the precursor in acetonitrile to the reactor vial |  |
| 05. Reacting at 130 °C for 10 min and evaporation of the solvent |  |
| 06. Addition of 1 M hydrochloric acid solution |  |
| 07. Evaporation of the solvent at 100℃ for 1min |  |
| 08. Addition of 1 M sodium acetate |  |
| 09. Injection on HPLC and collection of ^18^F-DiFA peak |  |
| 10. Trap product on QMA cartridge |  |
| 11. Elute product with 25% ascorbic acid (0.4mL) |  |
| 12. Evaporation of the solvent at 60℃ |  |
| 13. Formulation with 10mL saline |  |
| 14. Sterile filtration |  |
