# Supplementary material for: Biodistribution and radiation dosimetry of the novel hypoxia PET probe [18F]DiFA and comparison with [18F]FMISO
Source: EJNMMI Res. 2019 Jul 5;9:60. doi: 10.1186/s13550-019-0525-6 (PMC6611855; doi:10.1186/s13550-019-0525-6)

Supplementary File 2.

Wang W (1) and Thorwarth D (2) reported that the kinetic model of [^18^F]FMISO PET was a 3-compartment model (Figure). [^18^F]DiFA is also considered to be the same pharmacokinetic model as [^18^F]FMISO. The activity concentration at time of t (min) of plasma, the reversible compartment ( i.e. diffusion), [^18^F]DiFA trapped compartment, background ROI and lesion ROI are defined as C_in_(t), C_D_(t), C_A_ (t) ,C_bk_(t) and C_lesion_(t) respectively. Therefore, the next formulas are established.

$$C_{A}\left( t \right)=\frac{k_{1}k_{3}}{k_{2}+k_{3}}\otimes C_{in}\left( t \right)$$

$$C_{D}\left( t \right)=\frac{k_{1}k_{2}}{k_{2}+k_{3}}e^{-\left( k_{2}+k_{3} \right)t}\otimes C_{in}(t)$$

$$\otimes: convolution$$

$$C_{lesion}\left( t \right)=C_{A}(t)+C_{d}(t)$$

$$C_{bk}\left( t \right)=C_{D}(t)$$

When f and g are functions and a is scalar, the next formula is also established.

$$f\otimes\left( a\cdot g \right)=a\cdot(f\otimes g)$$

Because the amount of injected [^18^F]DiFA was very small, the dose and plasma concentration is in a linear relationship. Therefore, when an alfa-fold drug dose is administered, the activity concentration of plasma also becomes alfa-fold. The new lesion-to-background ratio is as follows;

$$C_{in}^{New}={\alpha\cdot C}_{in}^{Old}$$

$$Lesion-to-background ratio\left( New \right)=\frac{C_{lesion}^{New}\left( t \right)}{C_{bk}^{New}\left( t \right)};definition=\frac{C_{A}^{New}\left( t \right)+C_{D}^{New}\left( t \right)}{C_{D}^{New}\left( t \right)}=\frac{\frac{k_{1}k_{3}}{k_{2}+k_{3}}\otimes C_{in}^{New}\left( t \right)+\frac{k_{1}k_{2}}{k_{2}+k_{3}}e^{-\left( k_{2}+k_{3} \right)t}\otimes C_{in}^{New}\left( t \right)}{\frac{k_{1}k_{2}}{k_{2}+k_{3}}e^{-\left( k_{2}+k_{3} \right)t}\otimes C_{in}^{New}\left( t \right)}=\frac{\frac{k_{1}k_{3}}{k_{2}+k_{3}}\otimes{\alpha\cdot C}_{in}^{Old}+\frac{k_{1}k_{2}}{k_{2}+k_{3}}e^{-\left( k_{2}+k_{3} \right)t}\otimes{\alpha\cdot C}_{in}^{Old}}{\frac{k_{1}k_{2}}{k_{2}+k_{3}}e^{-\left( k_{2}+k_{3} \right)t}\otimes{\alpha\cdot C}_{in}^{Old}}=\frac{\alpha\cdot\left( \frac{k_{1}k_{3}}{k_{2}+k_{3}}\otimes C_{in}^{Old}+\frac{k_{1}k_{2}}{k_{2}+k_{3}}e^{-\left( k_{2}+k_{3} \right)t}\otimes C_{in}^{Old} \right)}{\alpha\cdot\left( \frac{k_{1}k_{2}}{k_{2}+k_{3}}e^{-\left( k_{2}+k_{3} \right)t}\otimes C_{in}^{Old} \right)}=\frac{\frac{k_{1}k_{3}}{k_{2}+k_{3}}\otimes C_{in}^{Old}+\frac{k_{1}k_{2}}{k_{2}+k_{3}}e^{-\left( k_{2}+k_{3} \right)t}\otimes C_{in}^{Old}}{\frac{k_{1}k_{2}}{k_{2}+k_{3}}e^{-\left( k_{2}+k_{3} \right)t}\otimes C_{in}^{Old}}=\frac{C_{lesion}^{Old}\left( t \right)}{C_{bk}^{Old}\left( t \right)}=Lesion-to-background raito(old)$$

Therefore, the lesion-to-background ration did not change when the administration dose was changed.

1. Wenli Wang, Jens-Christoph Georgi, Sadek A. Nehmeh, Manoj Narayanan, Timo Paulus, Matthieu Bal, Joseph O’Donoghue, Pat B. Zanzonico, C. Ross Schmidtlein, Nancy Y. Lee, John L. Humm. Evaluation of a compartmental model for estimating tumor hypoxia via FMISO dynamic PET imaging. Phys Med Biol. 2009 May 21; 54(10): 3083–3099.
2. Daniela Thorwarth, Susanne M Eschmann, Frank Paulsen and Markus Alber. A kinetic model for dynamic [18F]-Fmiso PET data to analyse tumour hypoxia. Physics in Medicine & Biology, Volume 50, Number 10:2209-24

Figure.


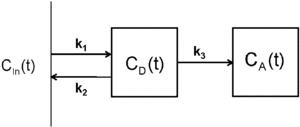

Supplement: Supplementary file 3 — The lesion-to-background ratio formula. (DOCX 30 kb) [file 13550_2019_525_MOESM3_ESM.docx]
