# Supplementary figures and images for: Biodistribution and radiation dosimetry of the novel hypoxia PET probe [18F]DiFA and comparison with [18F]FMISO
Source: EJNMMI Res. 2019 Jul 5;9:60. doi: 10.1186/s13550-019-0525-6 (PMC6611855; doi:10.1186/s13550-019-0525-6)

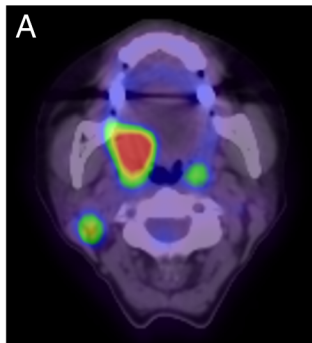

**C**

SUV  
2.5

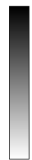

0.0

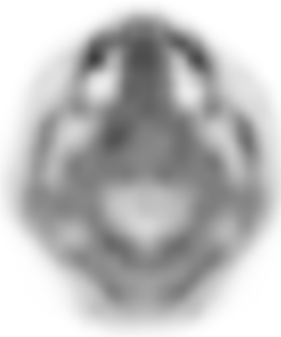

**E**

SUV  
2.5

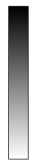

0.0

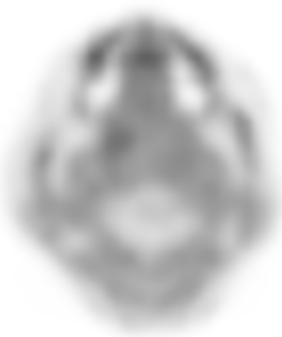

**B**

SUV  
6.0

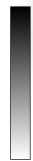

0.0

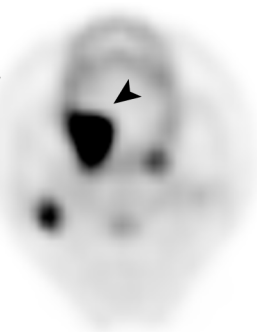

**D**

SUV  
2.5

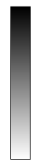

0.0

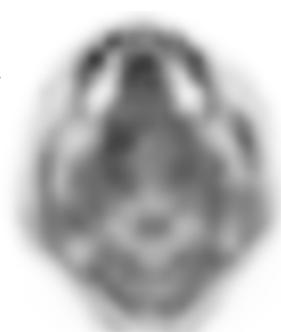

**F**

SUV  
2.5

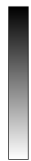

0.0

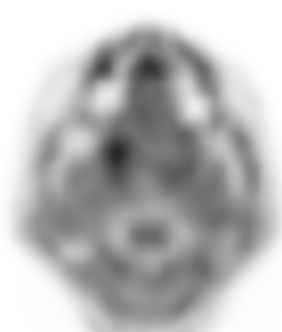

Supplement: Supplementary file 4 — Figure S1. [18F]DiFA vs. [18F]FMISO (PDF 736 kb) [file 13550_2019_525_MOESM4_ESM.pdf]
